# Postnatal corticosteroid therapy in bronchopulmonary dysplasia - why animal studies disagree with clinical trials?

**DOI:** 10.1038/s41390-024-03361-7

**Published:** 2024-06-24

**Authors:** Anantha Harijith, Thomas M. Raffay, Rita M. Ryan

**Affiliations:** Department of Pediatrics, Case Western Reserve University, 2109, Adelbert Road, Cleveland, OH 44106 USA

## Abstract

**Abstract:**

The systematic review and meta-analysis of newborn animal models by Irene Lok et al. is the first to extensively summarize the literature regarding postnatal systemic corticosteroid use on lung development of newborn rodent models. The meta-analysis showed that the use of postnatal corticosteroids resulted in a reduction in body weight along with persistent alveolar simplification. The most frequently used corticosteroid was dexamethasone. Corticosteroids have been extensively used in clinical trials in preterm newborns. Trials using early systemic administration of corticosteroids reduced the rate of BPD or mortality with no increase in the rates of cerebral palsy. Use of late systemic corticosteroids (administered >7 days after birth) also reduced the rate of BPD, mortality, and combined outcome of mortality or BPD. Late systemic corticosteroids showed no impact on the rates of neurodevelopmental outcomes in later childhood. It is important to note that later stages of inflammation leading to a more severe form of BPD continues to be a problem with no clear therapy in sight. The authors made a critical point in their paper – the negative effects of steroids were greater in the normal lung control animals than in the injured. This conveys caution in using steroids in a prophylactic manner.

**Impact:**

- Use of systemic corticosteroids in clinical trials have shown good response in preterm neonates evidenced by reduced rate of bronchopulmonary dysplasia.
- Rodent models have not shown a similar beneficial response.
- Use of systemic corticosteroids have caused greater arrest of lung development in rodent models with normal lungs compared to those with lung damage.

Corticosteroids are widely prescribed in the clinical practice of neonatology. While the use of corticosteroids to reduce the severity of neonatal preterm lung disease had declined following the American Academy of Pediatrics guidelines in 2002, postnatal steroid use seems to be regaining popularity in clinical practice.^1,2^ Animal models have been integral to our current understanding of neonatal physiology and the development of therapies in preclinical settings.

Up to 50% of infants born less than 28 weeks of gestational age develop bronchopulmonary dysplasia (BPD), a chronic lung disease of premature infants.^3^ Inflammation is considered a major cause of BPD.^4^ Over 50 years have passed since the first randomized controlled trial (RCT) of postnatal systemic corticosteroids to treat respiratory problems in preterm infants.^5^ However, the first randomized controlled trial of corticosteroids to specifically treat BPD was reported in the 1980s.^6^ The literature includes about 90 RCTs of corticosteroids to prevent or treat BPD, enrolling more than 9000 infants.^7^

This systematic review and meta-analysis of newborn animal models by Irene Lok et al. is the first to extensively summarize the current preclinical literature regarding postnatal systemic corticosteroids on lung development in healthy and diseased newborn rodent models.^8^ The meta-analysis showed that the use of postnatal corticosteroids resulted in a reduction in body weight along with persistent alveolar simplification. The most frequently used corticosteroid was dexamethasone (98%). In 73% of the rodent studies, corticosteroids were started in the first four postnatal days. A significant decrease in body weight was noted in the newborn rodents who were started on steroids before 15 days of age. This review included studies in both healthy newborn animals and those with hyperoxia-induced lung injury.

The current focus on the developmental evolution of BPD is the appearance of alveolar simplification, resulting in fewer but larger alveoli, producing less surface area for gas exchange. The authors demonstrate that corticosteroid treatment resulted in a reduction in the post-treatment number of alveoli as shown by reduced radial alveolar count (RAC) (Fig. 1), an increase in chord length (Lm), and a reduction in alveolar surface area in all steroid-treated newborn animals compared to non-steroid-treated controls. These measurements have been performed on a great many studies of steroid effects on the developing lung. Two possible explanations are offered by the authors for the absence of improvement following steroid treatment in RAC and Lm: (1) steroid-induced arrest of alveolar growth due to premature induction of maturation of lung cells; and (2) inability of steroid to dampen the impact of injury and inflammation caused by hyperoxia.^8^

**Fig. 1:**
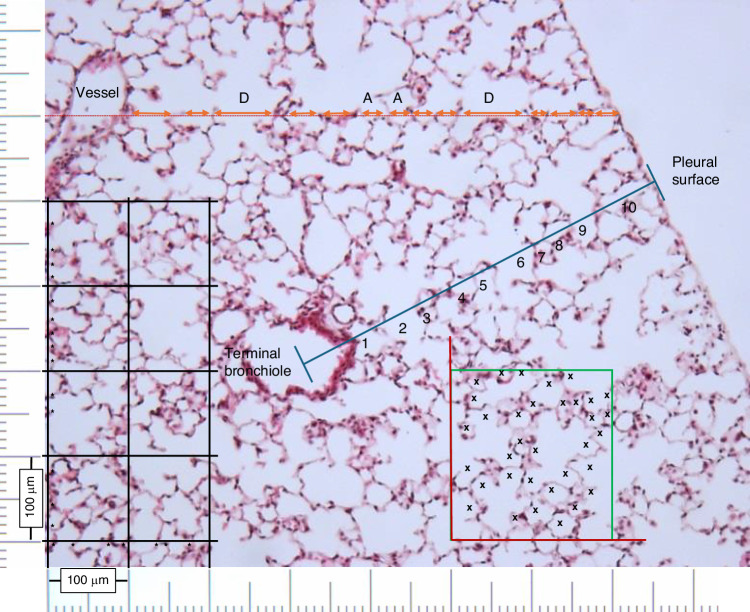
Measurement of alveolarization by Chord Length, Mean Linear Intercept and Radial Alveolar Counts. Chord Length – a test line is placed (dashed orange line with arrows) and the distance between alveolar structures that cross the line is measured, intercepts can span one alveolus (A), or cross the alveolar duct (D). Vessels are not counted. Mean Linear Intercept – 100 µm grid placed over lung fields (representative black lines), lung structures in the vertical and horizontal that intercept grid are counted (examples marked with asterisks), and the average linear distance between structures is calculated for the entire grid. Radial Alveolar Counts – a perpendicular line is drawn from a terminal bronchiole ending as a respiratory bronchiole to the lung interlobar septa (blue line) and the number of bisected saccules is counted (black numbers). Alveolar Counts – Alveoli structures are counted in sequential sections (not shown) within a given high-powered field with alveoli included (black X) if touching the inclusion line (green line) or excluded if touching the exclusion line (red line). For alveoli opening into alveolar ducts, a counting event is the presence of a bridge connecting the free edges of alveolar septae in the sampling section but not the sequential look-up section.

Studies administering postnatal corticosteroids to large (preterm) animals did not meet the inclusion criteria of this systematic review either because follow-up times were under 24 h or corticosteroids were not systemically administered. As introduced by the authors, it is possible that large animal models could better represent the multifactorial nature of BPD and could serve as better models to study the detrimental and beneficial effects of corticosteroids on lung development and BPD. It is clear that rodent animal models have not been successful in demonstrating the positive impact of steroid therapy that has been demonstrated in human clinical trials. This failure is partially attributed to the rapid lung development in rodents. For instance, a 10-day steroid treatment given to mice covers most of the saccular and almost half of the alveolar stage of lung development. This represents, physiologically, a long duration of treatment in preterm humans which clinically is never done. Administration of corticosteroid use in the first 10 days of postnatal life in a mouse would be equivalent to giving the drug from 24 weeks to almost a year of postnatal life in the human neonate/infant.^9^ Newborn rodents at birth are anatomically in the same late canalicular/ early saccular stage of lung development as a 24-week gestation preterm infant, but the rodent newborns are born at term and are not surfactant deficient but do resemble the state of a preterm infant who has received antenatal steroids to enhance surfactant production. Unfortunately, these differences preclude direct translation of pathology and timing of the postnatal steroid intervention in extremely preterm newborns to rodent models of BPD. Though rodents share similarities it is important to note that rats and mice are not interchangeable as animal models.^9^ The mice have smaller alveoli and have less lung parenchyma as a percentage of lung volume compared to rats.^10,11^ It may be that large animal models could help fill this gap in knowledge if the drug is administered for the duration corresponding to the same stage of lung development as in humans. This shortfall in rodent models also highlights the importance of the work of those who are creating human lung biorepositories or working to study respiratory function in preterm infants later in life. These efforts should be encouraged and supported in the future.

In this context, it is important to review the clinical use of corticosteroids in preterm neonates. Early systemic corticosteroids have been shown to be effective in reducing the rates of BPD at 36 weeks corrected gestational age (CGA).^12^ A reduction in the combined outcome of death and BPD was noted but not the mortality rates alone at 36 weeks CGA. Of note, most of the impact of early systemic corticosteroids in reducing rates of BPD has been demonstrated by dexamethasone rather than hydrocortisone.

Early administration of systemic corticosteroids (less than 7 days after birth) has been reported some time ago to increase the rates of cerebral palsy in later childhood. However, interesting data came from one of the most influential trials of early systemic corticosteroids, the PREMILOC trial, using hydrocortisone.^13,14^ Hydrocortisone reduced the rate of BPD or mortality at 36 weeks CGA, with no increase in the rates of cerebral palsy.^15^ In fact, neurodevelopmental outcomes were actually better for the 24–25-week gestation cohort in the hydrocortisone group. In general, as we are adding more studies with lower and shorter steroid dosing, the negative effect on the brain is no longer being noted. The use of late systemic corticosteroids (administered >7 days after birth) reduces the rate of BPD, mortality, and combined outcome of mortality or BPD.^12^ Similar to the early use of systemic corticosteroids, most of the effects of late systemic corticosteroids on BPD were with dexamethasone rather than hydrocortisone. Late systemic corticosteroids did not affect the rates of neurodevelopmental outcomes in later childhood.

The randomized clinical trial, SToP-BPD, studied the effect of systemic hydrocortisone, initiated between 7–14 days after birth in 372 infants.^16^ No significant difference in the composite outcome of death or BPD was noted between the hydrocortisone and placebo groups; however, there was significantly lower mortality in the hydrocortisone group, with resultant higher BPD. Another RCT of the effect of hydrocortisone on survival without BPD and on adverse neurodevelopmental outcomes at 22–26 months of age in intubated infants <30 weeks gestational age also showed no difference in death/BPD or neurodevelopmental impairment but did show a lower rate of remaining intubated in the hydrocortisone group.^17^ Recent meta-analyses of late (>7 days) starting of corticosteroids have continued to show benefit in reducing BPD without worse neurodevelopmental outcomes. In general, apart from a reduction in BPD, other beneficial effects of late systemic corticosteroids are lower rates of extubation failure and a smaller number of infants discharged home on oxygen.^12^ One of the major limitations of both the early and late systemic corticosteroid trials was that most of the studies were not powered to detect significant neurodevelopmental differences between the corticosteroid and control groups, but this has improved by using meta-analysis approaches.^18^

A knowledge gap perhaps still exists with the main finding of Lok et al. in terms of what alveolar simplification means to functional outcomes and lung/airway physiology (oxygen diffusion, exercise tolerance, susceptibility to respiratory infection, lung function studies) that are clinically important in the health outcomes of preterm children.^8^ In addition, later stages of inflammation leading to a more severe form of BPD continues to be a problem with no clear therapy in sight. Corticosteroids may offer a ray of hope for such patients.

In summary, though there are reasonable data to support the use of corticosteroids to prevent BPD, there are likely detrimental effects to the developing lung, as suggested by the current Lok et al. paper, from corticosteroids that we give to our NICU patients. The authors made a critical point in their paper – the negative effects of steroids were greater in the normal lung control animals than in the injured animals.^8^ It is possible that the dampening of inflammation by steroids has a greater positive effect than the negative effect on alveolar simplification for the relatively short-term BPD outcome. However, we do not know whether this is the case for later outcomes such as reactive airway disease, so commonly seen in NICU graduates, or even lung dysfunction much later in life.
